# Diagnostic Challenges of Traumatic Ulcerative Granuloma with Stromal Eosinophilia in the Hard Palate

**DOI:** 10.3390/diagnostics15020199

**Published:** 2025-01-16

**Authors:** Giacomo Setti, Stefania Caramaschi, Giuseppe Barile, Antonio d’Amati, Marta Forte, Giuseppe D’Albis, Pierantonio Bellini, Giovanna Garuti, Luisa Limongelli, Saverio Capodiferro

**Affiliations:** 1 Dentistry and Oral-Maxillofacial Surgery, Department of Surgical, Medical, Dental, and Morphological Science, University of Modena and Reggio Emilia, Via Del Pozzo 71, 41125 Modena, Italy; giacomo.setti@unimore.it (G.S.); g.garuti@unimore.it (G.G.); 2 Department of Medical and Surgical Sciences, University of Modena and Reggio Emilia, Via Del Pozzo 71, 41125 Modena, Italy; stefania.caramaschi@unimore.it; 3 Clinical and Experimental Medicine PhD Program, Department of Biomedical, Metabolic, and Neural Sciences, University of Modena and Reggio Emilia, Via Del Pozzo 71, 41125 Modena, Italy; 4 Department of Interdisciplinary Medicine, University of Bari “Aldo Moro”, 70121 Bari, Italy; giuseppe.barile@uniba.it (G.B.); giuseppe.dalbis@uniba.it (G.D.); luisa.limongelli@uniba.it (L.L.); saverio.capodiferro@uniba.it (S.C.); 5 Department of Precision and Regenerative Medicine and Ionian Area (DiMePRe-J), University of Bari “Aldo Moro”, 70100 Bari, Italy; antonio.damati@uniba.it

**Keywords:** TUGSE, oral cavity, oral pathology

## Abstract

Traumatic ulcerative granuloma with stromal eosinophilia (TUGSE) is a benign lesion that may arise from the oral mucosa consisting in an ulcerative lesion usually localized in the tongue or cheek. Palate localization is very rare. **Background**/**Objectives**: The aim of this study is to describe a case of TUGSE occurring in the hard palate of an 83 y.o. female patient, manifesting as a non-painful growing palatal lesion approximately of 3.5 × 2 cm with firm consistency and a central area of erosion and erythema, the difficulties in clinical diagnosis, and the criteria for the differential diagnosis. Also, considering the rare occurrence of TUGSE in the palate, we performed a review of the literature too. **Methods**: A comprehensive review of the literature was conducted following the 2020 PRISMA guidelines and a total of seven records were identified as matching the inclusion criteria of this study. **Results Conclusions**: Although TUGSE is a benign lesion, the clinical presentation as a proliferative and ulcerative lesion may be challenging for clinicians mainly when arising in rare sites of occurrence (e.g., the hard palate).

## 1. Introduction

Traumatic ulcerative granuloma with stromal eosinophilia (TUGSE) is a rare benign lesion of the oral mucosa, characterized by a rapid onset and a spontaneous regression which can generally last for several weeks. TUGSE manifestation was seen in two major distinct peaks; within the first two years of life, the disease is known as Riga–Fede disease and is mainly located on the anterior tongue/floor of the mouth and represents a reactive and self-limiting entity generally related to the trauma with primary dentition. The second peak of this bimodal age distribution of TUGSE occurs between the sixth and seventh decade [1,2]. No incidence difference between male and females was described (ratio 1:1) [3]. Etiopathogenesis is still unclear; viral and toxic agents could be involved, but trauma is generally accepted as the most frequent cause [4,5].

Despite being a well-characterized entity in common sites such as the tongue or cheek, TUGSE localized to the palate remains underreported [5,6]. Due to its clinical presentation as an ulcer, the rare palatal localization poses a diagnostic challenge for clinicians and pathologists, as it can resemble malignant oral diseases or infections. Differential diagnoses include conditions such as syphilis, tuberculosis, and Epstein–Barr virus mucocutaneous ulcer. Additionally, oral squamous cell carcinoma and lymphomas must also be considered [7].

Lesion development is probably due to the lack of TGF-a and TGF-b production by eosinophilic inflammatory infiltrate that significantly delays the mucosal healing [7]. Furthermore, some scientific evidence in the literature characterizes metformin as a TGF-b suppressor, supporting its association with TGF-b decrease and its ability to prevent TGF-b from attaching to its receptor [8].

The main histological features are the presence of a polymorphic inflammatory infiltrate with eosinophils and histiocytes, deeply involving the mucosal stroma as well as muscles and glandular tissue; pseudo-lymphomatous aspects are occasionally found as related to the high-rate mitosis as well as the presence of CD30-positive cells. Moreover, sometimes CD30+ cells may show monoclonal expansion with positive TCR γ rearrangement, which appears to be a confounding factor in the differential diagnosis with lymphoproliferative diseases [9].

Though the clinical appearance as a persistent ulcer could complicate the diagnostic–therapeutic work-up, an incisional biopsy with histological examination is mandatory and recurrence extremely rare [9]. In addition, surgical trauma after the biopsy procedure could promote the spontaneous healing of such entities [10,11,12].

Clinical course is extremely variable in terms of healing as it could last from days to months. Some studies report that lesions usually spontaneously resolve without the need of any treatment. Elimination of identified trauma is recommended [10].

The aim of this narrative review is to analyze the existing literature on TUGSE, with a particular focus on cases involving the palate, their clinical course, and treatment modalities. Additionally, a new case of palatal TUGSE is presented and discussed.

## 2. Materials and Methods

**Figure 1:**
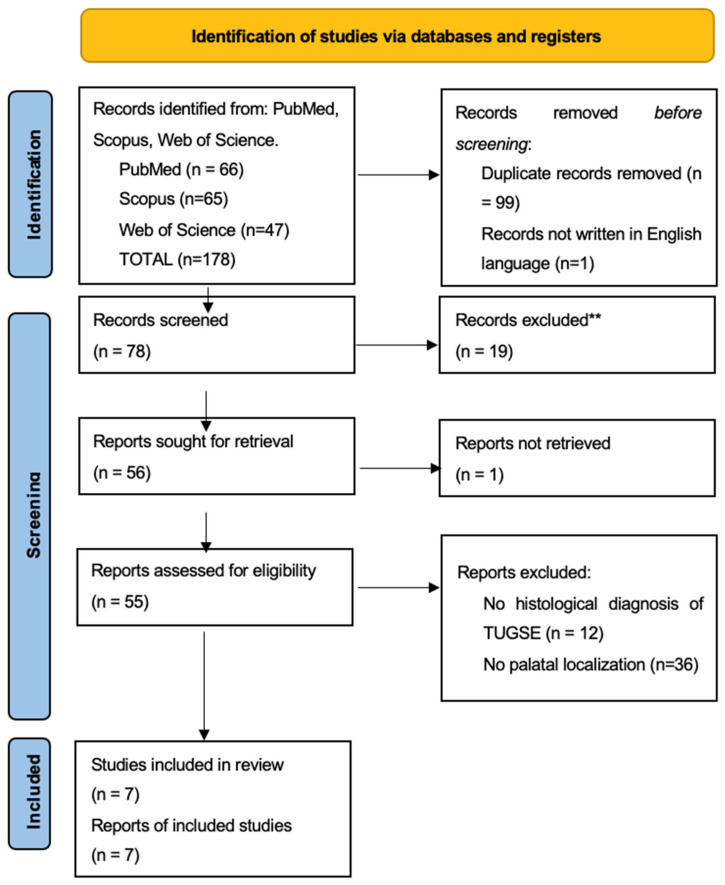
The 2020 PRISMA flow chart for reporting systematic reviews.

To perform this study, the authors conducted a comprehensive review of the literature regarding existing data about TUGSE; PRISMA guidelines were followed and are summarized in Figure 1. The terms “Traumatic Ulcerative Granuloma with Stromal Eosinophilia” OR “TUGSE” AND “oral” OR “oral cavity” OR “palate” were used and all kinds of paper in reliable databases (PubMed, Scopus, and Web of Sciences) were collected in the period from September 2024 to December 2024. Case reports, case series, reviews, and systematic reviews of the literature were included for the analysis. A total of 176 records was achieved following the application of the keywords to the databases. Only seven articles were included in this study; the authors excluded articles and studies where TUGSE histological diagnosis was not confirmed by examination and non-palatal localization of the lesion. Also, articles not written in the English language were excluded. Four independent reviewers (G.B., G.D.A., S.C. and M.F.) read, selected, and revised the included articles.

## 3. Case Presentation

An 83-year-old Caucasian woman was referred to the Dental and Oral-Maxillofacial Unit of the University of Modena and Reggio Emilia for the evaluation of a non-painful, long-lasting, and growing palatal lesion. The patient was a non-smoker, and no alcohol consumption was reported. The patient had a history of diabetes, atrial fibrillation, and dementia and she was in therapy with metformin, furosemide, quetiapine, tapentadol, venlafaxine, rivaroxaban, and potassium canreonate. In addition, an empiric therapy of 6 days of amoxicillin + clavulanate 1 g tabs twice-a-day had recently been prescribed by her general practitioner without any lesion modification or regression.

**Figure 2:**
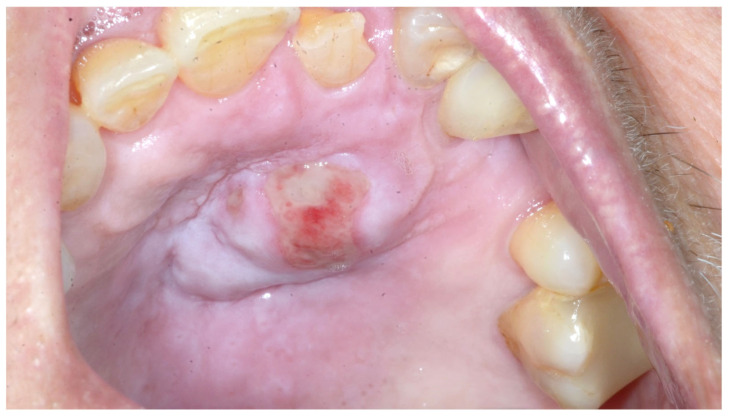
Ulcerated and firm swelling lesion of the hard palate with a central area of erythema.

At the intraoral examination, central incisor 21 presented a non-complicated coronal fracture without clinical and radiographic signs of pulp necrosis; no dental prosthetic devices were in use and no history of recent dental treatment was reported. Soft tissues were normal in the whole mouth except for a 3.5 × 2cm hard palate swelling of firm consistency, with a central area of erosion and erythema (Figure 2). Physical or chemical traumatic injuries were discussed with the patient and her relatives, considering the patient’s fragility related to dementia.

Extraoral examination was not significant; neck palpation did not allow the disclosure of enlarged cervical, supraclavicular, occipital, and pre-auricular nodes.

Differential diagnosis included: squamous cell carcinoma, minor salivary gland neoplasm, infectious diseases (e.g., tuberculosis; EBV muco-cutaneous ulceration), lymphoproliferative disorder, oral amelanotic melanoma, chronic eosinophilic ulceration, and possible trauma or chronic mutilation of which the patient was not aware. To achieve histological diagnosis, multiple punch biopsies were performed under local anesthesia in the most suspicious parts. No sutures were used. Interestingly, although epinephrine-free anesthetic was used for local anesthesia and injected only in the lesion periphery, no bleeding was observed during the incisional biopsy procedure. Further work-up included complete blood tests and neck nodes ultrasonography which returned no peculiar alterations.

**Figure 3:**
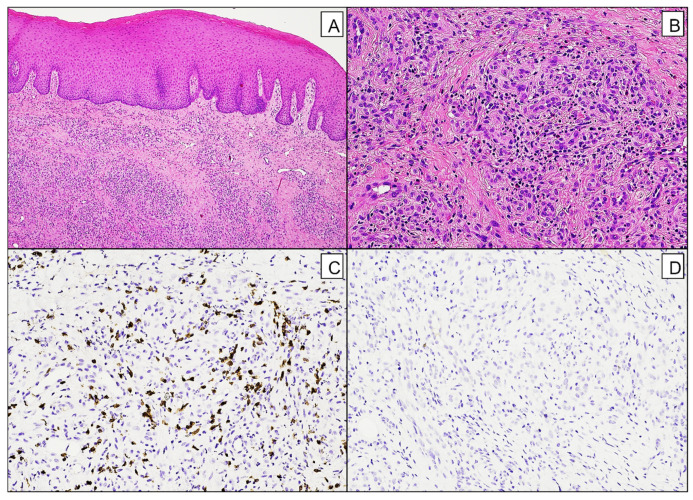
Histological findings. (**A**)—Oral mucosa with acanthosis and dense lympho-granulocytic infiltrate; H&E, 4x. (**B**)—Granuloma-forming inflammatory infiltrate composed of small round lymphocytes, eosinophilic and neutrophilic granulocytes, histiocytes, and plasma cells; H&E, 20X. (**C**)—Lymphoid infiltrate CD3+; IHC CD3, 20X. (**D**)—Lymphoid infiltrate CD30-; IHC CD30, 20X.

Histological examination described acanthosis, chorion lymphocytic and granulocytic infiltration with a high concentration of eosinophils. Inflammatory infiltration resembled a granuloma-forming pattern, with small round lymphocytes, eosinophilic and neutrophilic granulocytes, histiocytes, and plasma cells. Immunohistochemical investigations returned normal expression (+) of CD3, CD20, and ki67; CD30, HHV8, CKMNF116, p16, HPV, ERG, and S100 were all negative. No epithelial dysplasia was detected (Figure 3A–D).

**Figure 4:**
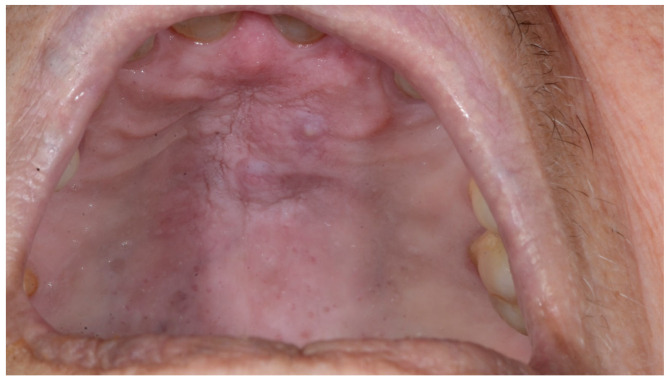
After 2 months, no sign of recurrence was observable.

At a 15-day follow-up, advanced remission of the lesion was observable, while complete (and always spontaneous) healing was achieved in about 2 months; no recurrence was detected at a 2-month follow-up (Figure 4).

## 4. Results

As for data from the literature, the current study analyzed seven articles published between 1960 and 2022, encompassing a total of seven cases of palatal TUGSE; demographic, clinical, and histological findings of palatal TUGSEs described in the literature are summarized in Table 1.

Clinically, the lesions were described as painful ulcerations in four cases, whereas one case was presented as a swelling. The diagnosis of TUGSE was confirmed in all cases through histopathological examination. Of these, four were males and three females with an age range of 41–77 y.o.; one case involved the soft palate, while the remaining six were localized to the hard palate.

Therapeutic approaches varied, with no treatment administered in two cases, corticosteroid therapy in one case, liquid nitrogen application in one case, and surgical excision in another. Among the cases with documented follow-up, complete resolution of the lesions was observed, with no reported recurrences.

Differently, the herein described case was diagnosed through multiple punch biopsies and subsequent histological examination, thus confirming the diagnosis of TUGSE. No additional treatment was administered. Complete healing of the site was achieved within two months, and no recurrences were observed during clinical follow-ups.

## 5. Discussion

Although the overall clinical course of TUGSE tends to be benign with a low rate of recurrences, the clinical presentation as a persistent ulcer often raises concerns of malignancy. In fact, the differential diagnosis includes a wide array of oral lesions with the same clinical presentation as a chronic, frequently unpainful, fast-growing ulcer, such as squamous cell carcinoma, lymphoproliferative disorder, infectious disease (mostly Epstein–Barr virus-related ones), primary syphilis, tuberculosis, and deep-seated mycoses, drug reactions, molluscum contagiosum, Kimura’s disease, Langerhans cell histiocytosis, sarcoidosis, lupus erythematosus (mostly discoid type), and Wegener granulomatosis [11,12]. Also, further difficulties in the differential diagnosis may arise in case of multiple TUGSEs, either synchronous or metachronous, also described in the literature [18].

In addition, as for the reported case, the rare palatal occurrence along with the peculiar clinical appearance as an ulcerated and developing lesion surely increases the plethora of differential diagnoses as well as the challenges for clinicians. Generally, the usually fast onset time of TUGSE may help clinicians in excluding oral squamous cell carcinoma, salivary neoplasm, syphilis, tuberculosis, or different diseases with a generally recognized slow growth; conversely, other reactive or inflammatory lesions may show a rapid onset like TUGSE [19,20,21]. Recognition of a potential (dental- or non-dental-associated) trauma surely remains the key factor for the differential diagnosis as well its early removal and the clinical monitoring at least for the following two weeks, which remains a median period for clinical re-evaluation and subsequent decision for the necessity of biopsy with histological examination.

To date, the etiology of TUGSE remains still debated, with trauma being a leading hypothesis. Riga in 1881 first described such lesions as usually occurring on the ventral surface of the tongue and associated to the traumatic action of the recently erupted mandibular incisors during the first two years of life [1,22]. In their respective studies, Marszałek et al. reported that trauma is present in less than 50% of lesions and Segura in 39%, respectively, so a strict correlation between trauma and TUGSEs development is still missing [1,23,24]. An interesting pathogenesis hypothesis is reported by Hirshberg et al.: a trauma may determine a mucosal breakdown where some unknown antigen may be occasionally permeated. Degranulating eosinophils emitting toxic chemicals or cytotoxic T-cell proliferations may be the cause of such mucosal degeneration. Aggregates of T-cell intracytoplasmic antigen 1+ cells and a cytolytic T-cell marker were present in every case, confirming the function of cytotoxic T-cells [18].

Common histological findings are present in the literature and generally described as an ulcer covered by a fibrino-purulent pseudomembrane; a rich granulation tissue with a heavy infiltrate of neutrophils, lymphocytes, plasma cells, and different grades of eosinophilic infiltrate CD30-positive large, atypical cells scattered or disposed in clusters compose the bottom portion of the ulcer [25]. The patient’s chronic inflammatory pattern involves the submucosa, and salivary glands, exactly as reported by Benitez et al., and deep muscle fibers if regarding buccal mucosa or tongue [26].

Histopathological findings, including the presence of CD30-positive cells, pose challenges in distinguishing TUGSE from lymphoproliferative disorders. CD30 large and atypical cells can be found in a wide range of lymphoproliferative disorders (LPDs) that includes cutaneous T-cell lymphomas, lymphomatoid papulosis, and anaplastic large T-cell lymphoma: it is a big family of benign disorders, usually indolent and with frequent spontaneous regression, where oral lesion share the same course of the cutaneous counterpart. Ficarra et al. suggested that TUGSE might represent the oral counterpart of the primary cutaneous CD30+ LPDs, while conversely Alobeid et al. believed that LPDs must not to be equated with TUGSE, which probably is a heterogeneous category of disorders [11,27].

A recent case report by Setti et al. describes some atypical TUGSE that displays CD30- cells with monoclonal rearrangement of the T-Cell Receptor γ chain gene, supporting a lymphomatous behavior. Conversely, an analysis of clonality for the T-cell receptor gene from Aizic et al. describes 100% of cases to be polyclonal for the TCR γ gene, remembering that there are neither cases of full lymphomatous transformation nor systemic involvement by T-cell lymphoma but only three cases of local manifestation of T-cell LPDs, supporting their reactive rather than neoplastic nature [10,28].

Despite the origin debate, in the literature only a small number of primary oral CD30+ T-cell lymphoproliferative disorders have been described: Rosenberg et al. described one case of such a lesion characterized by significant eosinophilic infiltration in a patient with a clinical history of a spontaneous healing oral mucosal ulcer [29]. Differential diagnosis is a difficult challenge for pathologists. Large, atypical cells that express bcl-6 have been reported as characteristic of anaplastic large T-cell lymphoma [26]. These cells are of histiocytic type, and the polymorphous inflammatory infiltrate rarely involves the underlying muscle in atypical histiocytic granuloma. In 1993, Regezi et al. discovered that these cells had either the dendrocyte marker factor XIIIa or the macrophage marker CD68; according to El-Mofty et al., the atypical large cells were only positive for vimentin but negative for of all lymphoid and histiocytic markers. This finding suggested that they may represent myofibroblasts and lately, in 2003, Cepeda affirms they originate from T lymphocytes [15,27,30,31,32].

Angio-lymphoid hyperplasia with eosinophilia is a rare condition involving the oral mucosa, typically manifesting as a nodule rather than an ulcer. Histopathologically, it is characterized by a prominent eosinophilic infiltration and the presence of bizarrely shaped blood vessels, without atypical monoclonal cells. Conversely, ulcerations associated with Langerhans cell histiocytosis are marked by the presence of atypical large cells of Langerhans cell origin, which are immunohistochemically positive for CD1a and exhibit ultrastructural Birbeck granules [12].

An angiodestructive pattern showing blood vessels with strong lumen reduction or complete vascular occlusion was described by our pathologist; this rare finding has been previously reported in a palatal TUGSE described by Brasileiro et al. [33].

Incisional biopsy is mandatory for definitive diagnosis when no evidence of spontaneous healing is observed after removal of potential etiological factors and a follow-up no longer than 2–3 weeks. After biopsy, spontaneous healing may occur, but a high variability could be observed, varying within 30 days up to 8 months [10].

Several therapeutical approaches have been proposed: wait-and-see, administration of nonsteroidal anti-inflammatory drugs, pain relief treatment with topical anesthetics, antibiotics, topical/intralesional or systemic corticosteroids, surgical curettage, cryosurgery [10,34]. Recurrences are infrequent but examples of intermittent representations have been also reported [9,24].

TUGSE occurrence on the palate is notably uncommon in contrast to the tongue and buccal mucosa, which are the most frequently involved sites [1,5,13,14,15,16,17]. This disparity may be attributed to underreporting and the lack of case series focusing on palatal presentations. This review seeks to address this gap by synthesizing the existing literature and highlighting critical diagnostic and therapeutic challenges. Furthermore, the observed heterogeneity in treatment approaches underscores the possible need for developing a standardized protocol for the management of TUGSE, particularly in rare and atypical localizations.

## 6. Conclusions

TUGSE, though benign, poses diagnostic challenges due to its clinical resemblance to malignancies. A multidisciplinary approach involving clinical, histopathological, and immunohistochemical analyses is essential for correct diagnosis and management. Further research is warranted to elucidate the etiopathogenesis and optimal therapeutic strategies for this rare oral lesion. Although its etiology is not fully understood, TUGSE generally follows a benign course with spontaneous healing. Most of the lesions resolve spontaneously but follow-up should be performed also in cases of complete remission.

## Figures and Tables

**Table 1:** TUGSE of the palate, literature review summarization.

Authors	Year	Sex	Age	Location	Clinical Description/Diagnosis	Histological/ Immunohistochemical Description	Treatment Other than Biopsy	Follow-Up
Knoth W. [13]	1960	M	53	hard palate	present from 8 months	Granuloma with eosinophils, simultaneosly skin lesions	NR	NR
R.P. Elzay [1]	1983	F	77	hard palate, located over a torus	NR	NR	excision	NR
Sklavounou A, Laskaris G. [14]	1984	M	42	hard palate, posterior	ulceration present from 3 weeks	eosinophilic and histiocitic infiltration	corticosteroids	healed in 1 month, no recurrences, follow-up NR
S.K. El-Mofty, P.E. Swanson, et al. [15]	1993	M	70	hard palate, right	6.5 × 3 × 1.8 cm swelling/NR	NR	NR	NR
H.S. Chung, N.S. Chung et al. [16]	1998	F	41	hard palate, left	Severe, painful 1 × 1 cm punched-out ulcer with erythematous base	widespread submucosal infiltration of inflammatory cells, primarily eosinophilic cells	liquid nitrogen	fast self-resolution, follow-up NR
F.P Fonseca, B.A. Benvenuto et al. [5]	2013	M	64	hard palate, right	painful single ulceration with no history of trauma/salivary gland tumor	CD30+, focal	no	no recurrences in 36 months
A. Shokravi, K. Ozcan et al. [17]	2022	F	51	soft palate, left	painful 2 × 3 cm ulceration/recurrent apthosus stomatitis	small lymphocytes, histiocytes, neutrophils, a large number of eosinophils, and sporadic plasma cells in a polymorphic inflammatory infiltrate	no	self-resolution in 3 months, follow-up NR
